# Letter to the editor: Urethrovesical anastomosis training: An innovative dual penile catheter approach

**DOI:** 10.1016/j.ijscr.2025.111215

**Published:** 2025-03-28

**Authors:** M. Tetou, M.A. Sobhi, M. Mrabti, A. Elbahri, A. Ameur, M. Alami

**Affiliations:** a Hassan II University of Casablanca, Faculty of Medicine, Morocco; b Mohamed V University of Rabat, Faculty of Medicine, Morocco

**Keywords:** Urethrovesical anastomosis, Surgical training, Urology, Penile catheter model, Laparoscopic surgery, Simulation

Dear Editor,

Urethrovesical anastomosis is a crucial stage in radical prostatectomy. Robot-assisted laparoscopic surgery makes it possible to perform this delicate procedure in a narrow space between the urethra and the bladder neck with great precision [1,2]. However, this procedure, performed at the end of the operation, becomes more difficult because of the fatigue accumulated by the surgeon [3].

Indeed, learning this technique by urologists can be difficult, notably because of the learning curve and the lack of accessible practical models [4,5]. In order to facilitate this training, we recently experimented with a simple and inexpensive method using two peniflow catheters, offering learners a realistic and repetitive training opportunity. This tip, which we wish to share with the urology community, has demonstrated a positive impact on technical mastery of urethrovesical anastomosis, particularly in a context of limited resources.

## 1 Methodology

### 1.1 Model setup

**Fig. 1:**
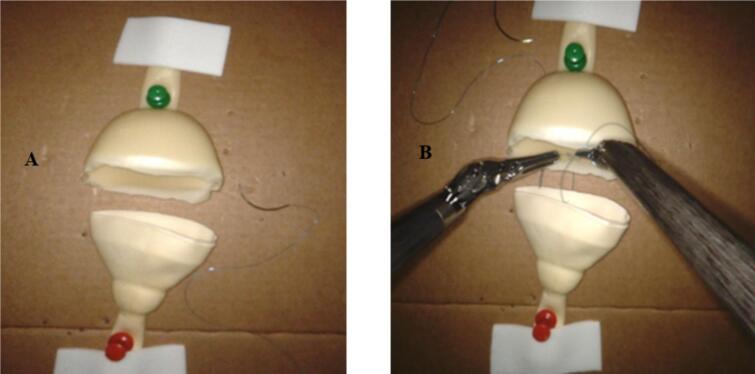
(A) A simulated urethrovesical anastomosis training model using two peniflow catheters. (B) The suturing of the posterior layer in the simulated urethrovesical anastomosis.

This training setup uses two Peniflow catheters to replicate the urethra and bladder (Fig. 1). Their latex composition and flexibility mimic the natural resistance of urethrovesical tissues, making the simulation both realistic and cost-effective. By providing a reproducible platform, this model allows surgical trainees to refine their suturing techniques in a structured and controlled setting.

### 1.2 Step-by-step assembly

#### 1.2.1 Bladder representation (vesical neck)

To replicate the bladder neck, a Peniflow catheter (outer diameter: 15–20 mm) is selected. A circular incision is carefully made at its tip, forming a vesical orifice (10–12 mm in diameter), which corresponds to the post-prostatectomy bladder opening seen in clinical practice.

#### 1.2.2 Urethral section simulation

A second Peniflow catheter is modified to represent the membranous urethra, the portion that remains following prostate removal. The distal end is cut and shaped to closely mimic the urethral sleeve, ensuring an accurate anastomotic site for training purposes.

#### 1.2.3 Secure attachment to base

Both components are firmly secured onto a stable, non-slip cardboard surface to ensure a fixed and realistic training experience.

The stabilization technique involves Small pins or tacks, which provide precise anchoring while avoiding damage to the latex material.

#### 1.2.4 Suturing simulation

Trainees engage in continuous suturing exercises, mimicking the posterior and anterior layers of a real urethrovesical anastomosis.

**Fig. 2:**
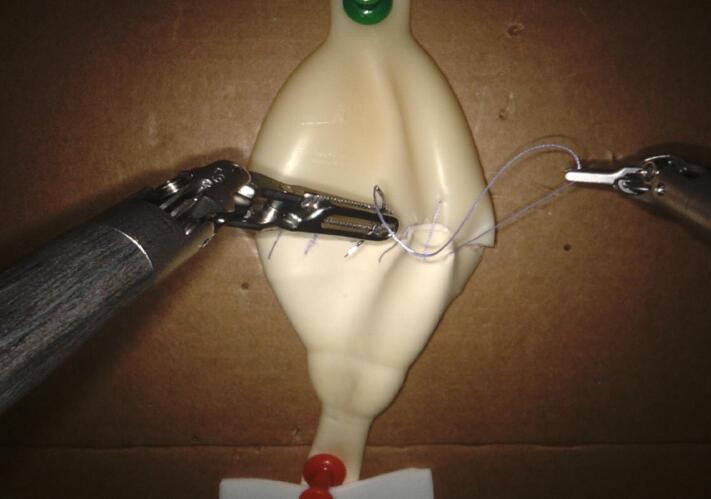
Completed urethrovesical anastomosis in the simulating model.

The following specifications are recommended:

‐ Suture type: 3-0 or 4-0 monofilament (e.g., polydioxanone or polyglecaprone).
‐ Needle selection: 17–19 mm curved needles, either reverse cutting or taper-point, to facilitate precise passage through the latex.
‐ Standardized suturing sequence:
  • Posterior layer begins from 4 o'clock to 8 o'clock, using either an interrupted or continuous suture pattern (Fig. 1).
  • Anterior layer: Final alignment of urethral and bladder edges is completed meticulously (Fig. 2).

## 2 Evaluation and feedback

Although we have not conducted a formal quantitative study, we collected structured feedback from surgeons and trainees in our department who used the model in practice sessions.

**Fig. 3:**
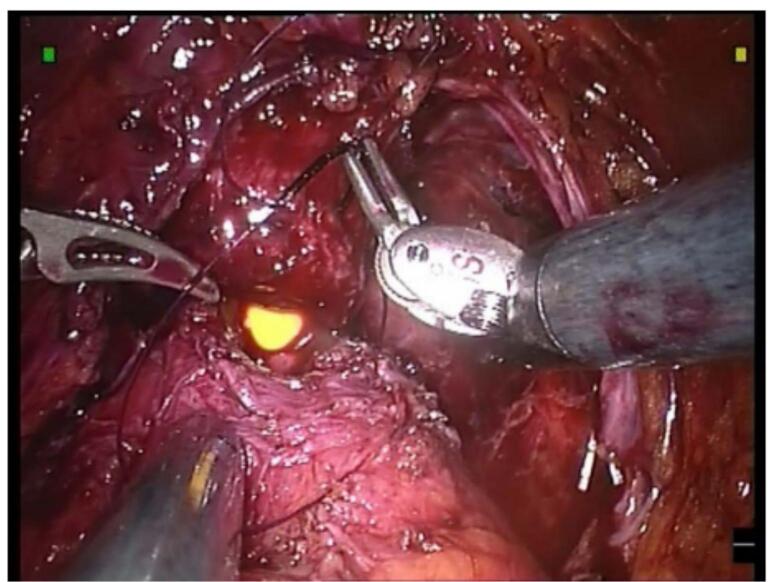
Peroperative image illustrating an urethrovesical anastomosis during a robot-assisted radical prostatectomy [6].
• 10 reported increased confidence in performing urethrovesical anastomosis.
• 8 noted improved suturing speed and precision after repeated practice.

• All participants (*n* = 15) found the model realistic and beneficial for training (Fig. 3).

This positive reception highlights the model's potential for skill acquisition and confidence-building among trainees. Future studies could integrate standardized assessment tools, such as the Visual Analog Scale (VAS) or validated simulation scoring systems, to formally evaluate its effectiveness.

## 3 Comparison with other training models

There is no universally accepted model for urethrovesical anastomosis training. Existing alternatives include:

• Synthetic tube models (e.g., millimeter-scale silicone or rubber tubing): These lack anatomical realism and do not accurately replicate tissue resistance.
• Cadaveric or animal models: While offering realistic tissue handling, these are costly, ethically restrictive, and require specialized facilities.
• High-fidelity robotic simulators: While beneficial, these are expensive and not always accessible in resource-limited settings.

## 4 Conclusion

Our dual catheter approach provides a simple, realistic, and affordable alternative that bridges the gap between accessibility and simulation fidelity.

## Author contribution

All authors have made substantial contributions to the manuscript as follows:

M. Tetou, MA. Sobhi, M. Alami: Concept and design of the training model, manuscript drafting.

M. Tetou, MA. Sobhi, M. Mrabti: Data collection and interpretation, manuscript revision.

M. Tetou, MA. Sobhi, M. Mrabti: Data analysis, manuscript editing.

M. Tetou, A. Elbahri, A. Ameur, M. Alami: Critical revision of the manuscript for important intellectual content.

All authors have reviewed and approved the final manuscript.

## Consent

This training approach did not involve direct patient participation or identifiable patient data. Therefore, individual patient consent was not required.

## Ethical approval

This study did not involve human participants or patient data; therefore, ethical approval was not required. The technique was developed and assessed in a simulated setting for educational purposes.

## Guarantor

M. Tetou.

## Research registration number

Our article focuses on a training model, not a research study, and therefore does not require registration of research studies.

## Funding

This paper did not receive any specific grant from funding agencies in the public, commercial or non-profit sectors.

## Conflict of interest statement

The authors of this paper declare that there are no conflicts of interest to disclose regarding the content of this manuscript.
